# Rewetting increases vegetation cover and net growing season carbon uptake under fen conditions after peat-extraction in Manitoba, Canada

**DOI:** 10.1038/s41598-023-47879-y

**Published:** 2023-11-23

**Authors:** Laurence Turmel-Courchesne, Marissa A. Davies, Mélina Guêné-Nanchen, Maria Strack, Line Rochefort

**Affiliations:** 1 Department of Plant Sciences, Université Laval, Québec, Canada; 2 Centre for Northern Studies and Peatland Ecology Research Group, Université Laval, Québec, Canada; 3 Department of Geography and Environmental Management, University of Waterloo, Waterloo, Canada

**Keywords:** Restoration ecology, Carbon cycle

## Abstract

The moss layer transfer technique has been developed to restore the carbon sequestration function and typical vegetation of *Sphagnum*-dominated peatlands after peat extraction in North America. However, the technique does not lead to successful bryophyte establishment when applied to peatlands with a richer residual fen peat. Therefore, we evaluated an alternative method of active rewetting and passive vegetation establishment using vegetation surveys and carbon dioxide and methane (CH_4_) flux measurements at a post-extracted fen in southern Manitoba, Canada. After one growing season post-rewetting, wetland vegetation established and the site was a net carbon sink over the growing season. However, high abundance of *Carex lasiocarpa* 10 years post-treatment led to higher CH_4_ emissions than the reference ecosystem. Successful establishment of wetland vegetation is attributed to the area being surrounded by undisturbed fens that can provide a local source of plant propagules. Bryophyte expansion was less successful than vascular plants, likely due to episodic flooding and shading from the sedge communities. Therefore, careful management of water levels to just below the peat surface is needed if reference vegetation community recovery is the goal of restoration. Water level management will also play a key role in controlling CH_4_ emissions to maximize carbon sequestration potential.

## Introduction

Peatlands play a significant role in the global carbon (C) cycle and climate regulation through their fluxes of carbon dioxide (CO_2_), methane (CH_4_), and dissolved forms of C^1^. These ecosystems store around a third of the earth’s soil C pool yet only cover approximately 3% of the land area^2,3^. Further, on centennial to millennial timescales, storage of C in peatland soils under waterlogged conditions counterbalances C released as CO_2_ and CH_4_, meaning peatlands have had a net cooling effect on Earth’s climate since their initiation^4–6^.

Conversely, drained peatlands are net C sources to the atmosphere and disproportionally contribute 5% of the global anthropogenic CO_2_ emissions from only 0.3% of the land area^7^. In North America, among other disturbances, some peatlands are drained to produce horticultural substrate through peat extraction. In order to extract the peat, vegetation is completely removed and the water level is lowered by drainage ditches^8,9^. Site preparation and extraction activities subsequently induce important changes in the biogeochemical functioning of peatlands, turning them into large CO_2_ sources^10–12^, while CH_4_ emissions are considerably reduced due to the lowering of the water levels, although ditches remain CH_4_ sources^13^. After extraction activities have ceased, extracted peatlands differ from undisturbed peatlands in that they have high soil temperatures, caused by the dark coloration of the peat, and faster rates of decomposition due to aeration^14,15^. Bare peat is also exposed to frost heaving, a form of substrate instability caused by the formation of ice needles that causes physical damage to vegetation trying to establish^16,17^. Therefore, peatlands can stay devoid of vegetation for decades after extraction and be colonized by vegetation assemblages not typical of natural peatlands^18,19^. Consequently, active restoration methods are often needed to promote the return of vegetation cover typical of peatlands and their C sink potential^20,21^.

One established restoration technique in extracted peatlands, called the moss layer transfer technique (MLTT), has been shown to successfully restore the plant community and C sequestration function in *Sphagnum*-dominated peatlands through profiling, introducing vegetation from a donor peatland, fertilizing, and rewetting^20–27^. However, when the residual peat of an extracted area is characterized by the physiochemical properties of minerotrophic fens that have higher pH, electrical conductivity (EC), and nutrient content than ombrotrophic bogs, vascular plants have been shown to establish and expand more successfully than bryophytes with mechanical re-introduction^28–31^. Therefore, alternative restoration techniques may be needed to establish comparable vegetation cover to reference communities, especially since bryophytes are likely important for the promotion of long-term C sequestration in peatland soils^32–34^.

Previous work on fen restoration has shown that wetland and peatland vegetation may be able establish and expand passively (i.e. without active reintroduction) when wet hydrological conditions are re-established^29,31,35–38^. However, CH_4_ and CO_2_ exchange is highly dependent on the vegetation cover and the hydrological conditions at the site, suggesting that there are potentially trade-offs between promoting the return of reference vegetation communities and C sequestration function between passive versus active vegetation establishment techniques^21,30,31,39^. Further, passive establishment of vegetation does not necessarily lead to bryophyte establishment, suggesting that a combination of techniques may be needed depending on local conditions, including if there is a local source of propagules^29,40,41^. Therefore, this study aims to (1) test whether rewetting without active planting of propagules is sufficient to establish typical fen vegetation communities when the site is surrounded by a reference fen ecosystem and subsequently, (2) evaluate how passive vegetation treatments impact CO_2_ and CH_4_ fluxes at a post-extracted site with residual fen peat in southeastern Manitoba, Canada.

## Methods

### Site description

**Figure 1:**
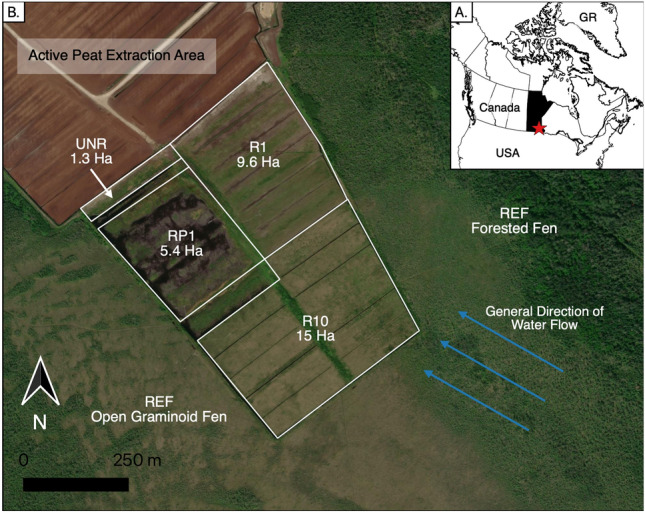
(**A**) Location of restored fen site in southeastern Manitoba, Canada. The province of Manitoba is in black and study location is marked by the red star. *GR* Greenland. (**B**) Location of the experimental sectors and reference sites. *UNR* unrestored sector, *RP1* one growing season after rewetting and profiling sector, *R1* one growing season after rewetting sector, *R10* ten growing seasons after rewetting sector, *REF* reference site. Base map from ESRI World Imagery (Maxar (Vivid) Imagery, 1 pixel = 0.5 m, August 1, 2018). Map was created by M. Davies using QGIS software^45^.

The study area is located within a large peatland complex extending over several thousand hectares in southeastern Manitoba, Canada and the Boreal Shield Ecozone^42^ (49.931° N, 96.237° W; Fig. 1). Mean annual temperature is 2.8 °C and total annual precipitation is 578 mm at a nearby weather station (Pinawa; approximately 30 km from the site; 1981–2010 climate normal^43^). The highest temperatures occur in July (19.3 °C) and the lowest in January (− 16.6 °C^43^). Snowfall > 10 cm occurs for 5 months of the year (November to March^43^). The 2016 growing season, in which measurements for this study were taken, was characterized by above average precipitation (i.e. 28% higher than the 1981–2010 normal; May to September^43,44^). Consequently, water levels measured in this study were likely higher than those of a typical growing season.

The study area is split into two broad regions: the reference site and the experimental sectors. The reference site (REF) is an open graminoid to forested rich fen located close to the experimental sectors (Fig. 1, Supplementary Fig. 1 and Table 1). The forested portions of the reference site are characterized by *Larix laricina* and *Betula pumila* as the main tree and shrub species, while the more open graminoid portions are dominated by *Carex lasiocarpa*. Bryophyte cover is over 50% on average within REF and is predominately *Campylium stellatum, Scorpidium cossonii,* and *Scorpidium scorpoides.* The experimental sectors cover an area of 35 ha and are located at the southeastern tip of a 237-ha peat extraction site (Fig. 1). The depth of the residual peat layer is greater than 1 m over the whole area and the peat and pore waters had a pH typical of poor to moderately rich fens^46^ (Supplementary Table 1). The experimental site was further divided into four sectors that correspond to different restoration actions and the number of years elapsed since the restoration. Those four sectors are: (1) after peat extraction but without restoration actions (unrestored; UNR), (2) after peat extraction and one growing season after rewetting and profiling (RP1), (3) after peat extraction and one growing season after rewetting (R1), and (4) after peat extraction and ten growing seasons after rewetting (R10). These four sectors plus the reference site (REF) make up the five measurement locations in this study (Supplementary Fig. 1). Rewetting was done through ditch blocking and when profiled, berms were created.

### Carbon dioxide and methane fluxes and environmental variables

Methane and CO_2_ fluxes were measured every one to two weeks at the experimental and reference sites to assess the impact of rewetting on C exchange post-extraction. Fluxes were measured between May 24 and September 14, 2016. A flux with negative value is considered an uptake of C into the ecosystem, while a positive value indicates C loss to the atmosphere. A total of six to nine steel collars (60 × 60 cm) were installed at the beginning of May 2016 in each experimental sector and the reference site to represent the major vegetation cover types and have replicates (i.e. bare, open water, herbaceous, and shrubs). After each CO_2_ and CH_4_ flux measurement, water table level was measured inside a well. Wells were located less than 1 m from each collar. The soil temperature profile was also measured every 5 cm down to a depth of 20 cm with a thermocouple thermometer.

The closed chamber technique was used to measure both CO_2_ and CH_4_ fluxes. Carbon dioxide measurements were performed by placing a clear acrylic chamber (60 × 60 × 30 cm) equipped with a thermocouple and a battery-operated fan for 2–3 min on the installed collars. Over that period, CO_2_ concentration was recorded every 15 s using a portable infrared gas analyzer (IRGA; EGM-4, PP Systems, Massachusetts, USA). Net ecosystem exchange (NEE) was determined using the linear change in concentration of CO_2_ in the chamber over the measurement period, corrected for the air temperature recorded at the time of sampling and volume of the chamber. Carbon dioxide fluxes were measured under different light levels, created using a series of shades and assessed with a photosynthetically active radiation (PAR) sensor installed on top of the chamber. Completely dark conditions (PAR = 0) were used to determine ecosystem respiration (ER). Gross ecosystem productivity (GEP) was calculated as the difference between NEE and ER. Carbon dioxide fluxes with stable concentration over the measurement period (< 2 ppm) were assumed to be equal to zero. Aside from these zero fluxes, non-linear fluxes (slope r^2^ ≤ 0.75) were deleted from dataset resulting in 14% data loss for *RP1* (see further methods for this site below) and less than 1% for the other measurement locations.

Methane flux measurements were performed on the same steel collars using an opaque acrylic chamber (60 × 60 × 30 cm). Samples were collected from the closed chamber after 7, 15, 25, and 35 min and stored in pre-evacuated vials (Exetainers, Labco Ltd., UK). Methane concentration in each vial was measured with a Shimadzu GC-2014 gas chromatograph (GC) equipped with a flame ionization detector at the University of Waterloo, Canada. The GC was calibrated with 1, 5 and 50 ppm standards with weekly checks for calibration drift. Methane flux was calculated from the linear change in concentration over time corrected for air temperature measured at the time of sampling and the volume of the chamber. Fluxes with a slope close to zero and concentration change within the precision of the sampling and analysis method (0.5 ppm) were considered equal to zero. Inconsistent fluxes suggesting ebullition (slope R^2^ < 0.60) were removed from the data set, inducing 7% data loss.

At RP1, spring rainfall and snowmelt combined with both rewetting and profiling the surface resulted in very high water levels over the entire measurement period (i.e.  > 38 cm above the surface on average; see Supplementary Table 2). Therefore, only CH_4_ and ER fluxes were measured by inserting opaque chambers onto submerged collars embedded into a boardwalk structure. ER fluxes were determined by measuring CO_2_ concentration in gas samples collected for CH_4_ flux with a Shimadzu GC-2014 gas chromatograph using a thermal conductivity detector at the University of Waterloo, Canada. Standards of 100, 300 and 500 ppm were used for calibration and with weekly checks for calibration drift.

Total growing season CO_*2*_ exchange was estimated using empirical models for GEP and ER for each collar. GEP models were based on the relation between GEP and PAR values from the series of shades at each collar using a rectangular hyperbola (after Strack et al.^47^):

1 $$GEP=\frac{PAR\times Q\times {GP}_{max}}{(PAR\times Q+{GP}_{max})},$$

where Q is the quantum efficiency and GP_max_ is the theoretical maximum GEP rate that represents the initial slope and asymptote of the hyperbola respectively. Values for Q and GP_max_ were calculated for each collar by minimizing the difference between observed and predicted GEP values. Depending on the available data and best fit, Q and GP_max_ values were determined for two or three time periods: early summer (May–June), mid-summer (July), and late summer (August–September) or a combination. Total GEP for the growing season for each collar was then calculated using the Q and GP_max_ values from each collar and hourly PAR values across the study period measured with a LI-190 (LI-COR, Nevada, USA) connected to a Campbell Scientific CR1000 data logger at a weather station in center of the experimental site and calibrated against the PAR sensor used during chamber flux measurements.

ER models were based on the relationship between ER and air or soil temperature at 5 cm depth (T_air_ or T_5_) and/or and water table level (WTL) depending on best fit, using either a multiple linear regression or exponential relationship:

2 $$ER=a\left({T}_{air}\mathrm{or }{T}_{5}\right)+bWTL+c,$$

3 $$ER= {ER}_{ref} \times \mathrm{exp}({E}_{0}\left[\frac{1}{{T}_{ref}-{T}_{0}}-\frac{1}{{T}_{air}- {T}_{0}}\right]),$$

where the parameters *a*, *b*, and *c* in Eq. (2) and ER_ref_ and E_0_ in Eq. (3) were fitted to minimize the difference between observed and predicted ER for each collar. T_ref_ and T_0_ were set to 10.35 °C and − 35.67 °C respectively, where T_ref_ represents the temperature at which ER_ref_ occurs and T_0_ is the lower temperature limit at which biological activity starts, with values chosen according to Günther, et al.^48^. Temperature values for Eq. (3) are converted to Kelvin prior to the calculation. Total ER for the growing season for each collar was then calculated using hourly temperature (T_air_, T_5_) and WTL for the model recorded near the collars using an Onset HOBO Pro v2 and Solinst levelogger, respectively for each site. Average hourly values for GEP and ER were summed to estimate the total at each collar over the 113-day study period (hereafter referred to as the growing season).

Total growing season CH_4_ flux for each sample collar was estimated by linear interpolation between measurements using the following equation (after Green and Baird^49^):

4 $${F}_{g, 1-2}= \frac{1}{2}\left({f}_{g,1}+{f}_{g,2}\right)\left({t}_{2}-{t}_{1}\right),$$

where ${F}_{g, 1-2}$ is the integrated CH_4_ flux between a pair of instantaneous fluxes or measurements (${f}_{g}$) at Time 1 and Time 2 (${t}_{1}$,${t}_{2}$). The ${F}_{g}$ values were added together to give the total CH_4_ flux at each collar. As for the GEP and ER estimations, CH_4_ linear interpolations were based on the 113-day study period. Total modelled CH_4_ flux was converted to a CO_2_ equivalent (i.e. 27 × the global warming potential of CO_2_ for a time horizon of 100 years for non-fossil CH_4_^50^). The methane CO_2_ equivalent was added to the modelled NEE values (GEP minus ER) to estimate global warming potential for each collar.

### Vegetation classification and community characterization

Vegetation surveys were conducted in August 2016 and 2017 to assess changes in vegetation community composition as the result of rewetting and profiling treatments. At the experimental site, surveys were performed in each sector along 50 m transects parallel to the drainage ditches. The starting position of each transect was randomly selected along the length of the peat fields. The number of transects was proportional to the surface area in each sector. At the reference site, three areas within the open graminoid and forested fen were surveyed for a total of 6 transects. Along each transect, vegetation was evaluated in five equally distant plots. Inside each plot, the proportion (%) of the surface covered by vascular plants (one 1 × 1 m quadrat) and bryophytes (ten 30 × 30 cm quadrats) was assessed by vertical projection. Because of high water levels, no vegetation surveys were completed at R1P.

The vascular plant and bryophytes species identified in the vegetation surveys were also placed into four preferential habitat categories (peatland species; wetland species, wetland facultative species and other species) following a methodology adapted from Poulin, et al.^22^. Peatland species are preferentially found in peatlands (*Sphagnum* peatlands or fens). Wetland species can be found in peatlands (but not preferentially), as well as other types of wetlands (e.g. marshes). Wetland facultative species can be found in wetlands, but not preferentially. Other species are not usually found in wetlands, but in other types of ecosystems like uplands. Vascular plant species were mainly categorized following Jeglum^51^, Payette and Rochefort^52^, and Gignac, et al.^53^. Bryophytes species were mainly categorized following Payette and Rochefort^52^, Faubert^54^, and Vitt^55^.

In addition to the sector-scale vegetation surveys, vegetation communities associated with the collars were also characterized through percent cover of plant groups and vegetation volume index. Vegetation surveys in the collar were completed at the end of the study period to match the timing of the sector-scale surveys (August 2016). Each collar and each vegetation plot from the whole site vegetation surveys was assigned to one of the four major vegetation categories to scale collar fluxes to the sector scale (i.e. bare, open water, herbaceous, and shrubs). Non-open water plots were considered bare when vascular vegetation was < 20% and were considered shrub dominated when shrub cover was > 20%. The rest of the plots were considered herbaceous (see Supplementary Fig. 2). The relative abundance of each plot type was then used to calculate weighted means of the total NEE, GEP, and ER for each sector from the fluxes for the matching collar types.

Vegetation volume index was measured coincident with the flux measurements at each collar following the methodology described by Davies, et al.^56^. To measure vegetation volume index, a stick painted in white and red bands was placed vertically in the collars and the proportion (%) of each band obscured by vegetation as well as the proportion (%) of vegetation cover in the collars was recorded (i.e. bryophyte and vascular species). Five measurements inside individual collars were made each time and averaged. Obstruction values were then transformed to vegetation volume index using *PObscured* (www.firebeaters.org.uk).

### Evaluation of factors controlling methane and carbon dioxide fluxes

To investigate the controls on CO_2_ and CH_4_ fluxes and the differences between the experimental sectors and the reference site as the result of rewetting, linear mixed models were built using combinations of air/soil temperature, vegetation volume index, and water table position as fixed factors, using the *nlme* package in R^57,58^. Sector and portion of the study period were also tested as fixed factors in a separate model to test whether site location influenced the response to changes across the growing season (i.e. periods of May–June, July, and August–September respectively). One model was built for each C flux component (ER, GEP, NEE, CH_4_). Methane data was transformed prior to analysis to improve normality (i.e. log_10_(CH_4_ + 15)). A random factor was included to account for repeated measurements on individual collars over the study period. The study location was used as a grouping factor inside the models that tested the impacts of environmental variables on each C flux component to account for heterogeneity of the variance between sectors (*varIdent* function of *nlme* package^58^). In all cases, NEE and GEP used in this investigation included only values in which PAR photon flux density was greater than 1000 µmol m^–2^ s^–1^, representing rates when PAR is saturated^59^. Because of very high water tables and absence of vegetation at *RP1*, specific linear mixed effect models were built to investigate controls over CO_2_ and CH_4_ fluxes for that sector. For *RP1*, water table position (i.e. water level) and soil and air temperature were used in mixed models as fixed factors. Models were visually inspected for normality and homogeneity of the residuals and possible leverage effect. When a factor significantly explained variation in the data, Tukey pairwise comparisons were executed in order to evaluate differences in CO_2_ and CH_4_ fluxes between sectors (*lsmeans* package^60^). A significance level of α = 0.05 was used for all tests.

## Results

### Vegetation community structure

**Figure 2:**
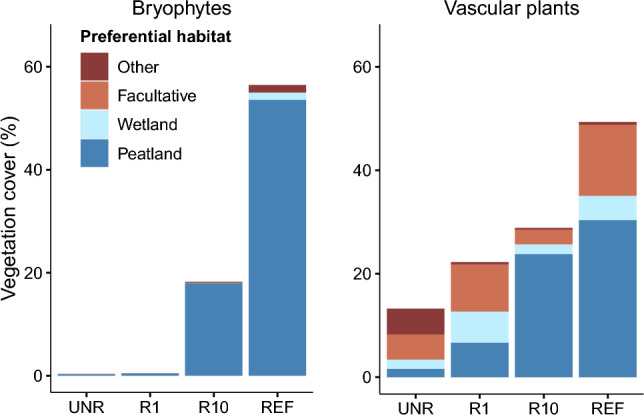
Mean cover (%) of bryophytes and vascular species for each sector. Species are classified according to their preferential habitat. Peatland: species preferentially found in peatlands (*Sphagnum* peatlands or fens), Wetland: species preferentially found in wetlands, Facultative: species that can occur in wetlands, but not preferentially, Other: species preferentially found in other types of ecosystems (e.g. upland ecosystem). *UNR* unrestored, *RP1* 1 year after rewetting and profiling, *R1* 1 year after rewetting, *R10* 10 years after rewetting, *REF* reference site.

Total vascular vegetation cover was highest at the reference site (REF) and lowest in the unrestored sector (UNR; Supplementary Table 3). Vascular vegetation one year after restoration (R1) reached 26% on average, was mostly herbaceous, and was composed of a mix of peatland and wetland species as well as species not preferentially found in wetlands or peatlands (Fig. 2 and Supplementary Table 3). At 10 years after restoration (R10), vegetation was predominantly composed of the peatland species *Carex lasiocarpa* (Supplementary Table 3). UNR was dominated by species not preferentially found in wetlands or peatlands, mainly the ruderal species *Hordeum jubatum* and *Agrostis scabra* (Supplementary Table 3 and Fig. 2).

Total bryophyte cover was also highest at REF and lowest at UNR, with average covers of 57 and < 1%, respectively (Supplementary Table 3). One year after restoration (R1), bryophyte cover remained low and comparable to UNR. Although R1 had a comparable bryophyte cover to UNR, a greater number of peatland species were present (Fig. 2 and Supplementary Table 3). At 10 years since restoration (R10) mean bryophyte cover had increased to 18% (Supplementary Table 3). Both R10 and REF had the same dominant bryophyte species: *Campylium stellatum* and *Scorpidium cossonii*. However, *Sphagnum* species only had < 1% coverage at R10, while covering on average 6% at REF (Supplementary Table 3).

### Carbon exchange and controlling factors

**Figure 3:**
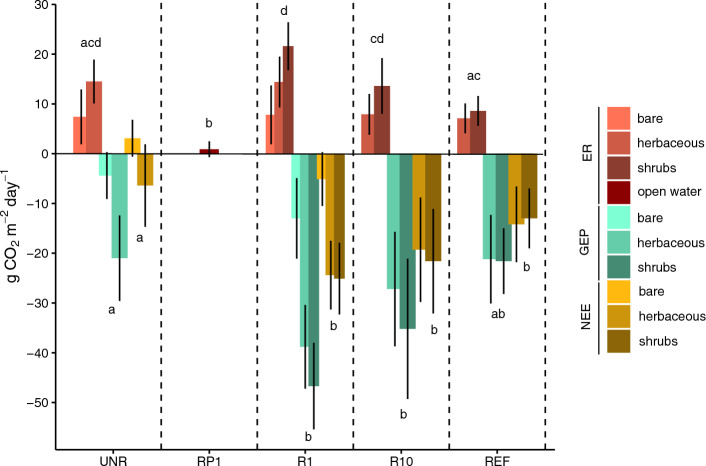
Mean (± SD) ecosystem respiration (ER), gross primary productivity (GEP) and net ecosystem exchange (NEE) for each major vegetation group across the entire study period (May–September 2016) at an extracted fen in southeastern Manitoba, Canada. GEP and NEE are measured at photon flux density of photosynthetically active radiation greater than 1000 μmol m^–2^ s^–1^. Negative values represent uptake by the ecosystem. Bar groups sharing a letter are not significantly different (Tukey pairwise comparisons, α = 0.05). Results are not sorted according to period of the growing season (May–June, July, and August to September) because there was no significant interaction between sector and period for NEE and GEP linear mixed effects models (see Supplementary Table 4). Letters should be compared only within one flux component. Error bars represent the standard deviation of the means for each vegetation group. Least squares means and 95% confidence intervals for NEE, GEP, and ER for each bar group are found in Supplementary Table 5. *UNR* unrestored, *RP1* 1 year after rewetting and profiling, *R1* 1 year after rewetting, *R10* 10 years after rewetting, *REF* reference site.

Mean daily and total seasonal NEE values were generally negative within each sector and the reference site, with greater CO_2_ sequestration occurring in the restored (R1, R10) than in the unrestored sectors (UNR; Fig. 3 and Table 1). The exception was the bare collar locations within UNR, which had the lowest vascular plant coverage and a daily positive NEE (Table 2 and Fig. 3). As UNR had a high percentage of the bare cover type, it was a net C source over the growing season (Table 1). Mean and total seasonal ER values were generally similar across sectors, but greater vegetation cover at restored sites compared to UNR led to greater GEP, and hence greater CO_2_ uptake in response to rewetting (Fig. 3 and Table 1). The exception was the sector that had high water levels for the duration of the study (RP1) that had lower ER and lacked vegetation cover (Fig. 3 and Table 2).

**Table 1:** Mean (± SD) net ecosystem exchange (NEE), net methane exchange (CH_4_), carbon balance (C) and global warming potential (GWP) calculated over the study period (113 days) for each sector and major vegetation cover type.

Sector	Cover type	# Chambers	NEE (g CO_2_ m^–2^)	CH_4_ (g CH_4_ m^–2^)	Total C (g C m^–2^)	GWP (g CO_2_-e m^–2^)
UNR	Bare (82%)	3	170 (69)	0.3 (0.2)	47 (19)	177 (73)
	Herbaceous (18%)	3	− 181 (66)	1.9 (1.6)	− 48 (18)	− 130 (77)
	ALL (weighted mean)	6	107 (68)	0.5 (0.5)	30 (19)	122 (73)
RP1	Open water (100%)	6	138 (108)	8.7 (8.7)	44 (30)	373 (258)
R1	Bare (15%)	3	− 102 (231)	9.4 (8.6)	− 21 (62)	152 (292)
	Herbaceous (66%)	3	− 1175 (255)	11.0 (6.9)	− 312 (71)	− 879 (354)
	Shrubs (19%)	3	− 1061 (235)	7.3 (8.1)	− 284 (64)	− 864 (305)
	ALL (weighted mean)	9	− 992 (248)	10.0 (7.4)	− 263 (68)	− 722 (335)
R10	Herbaceous (98%)	3	− 1053 (323)	26.8 (10.2)	− 267 (80)	− 329 (53)
	Shrubs (2%)	3	− 1437 (318)	33.3 (6.4)	− 367 (87)	− 539 (358)
	ALL (weighted mean)	6	− 1060 (322)	26.9 (10.1)	− 269 (81)	− 333 (59)
REF	Shrubs (47%)	5	− 493 (371)	6.6 (4.2)	− 130 (100)	− 315 (319)
	Herbaceous (53%)	4	− 683 (483)	8.6 (5.6)	− 180(130)	− 452 (425)
	ALL (weighted mean)	9	− 594 (430)	7.6 (4.9)	− 156 (116)	− 388 (376)

Cover of each type is weighted by site level vegetation survey plot classifications.

*UNR* unrestored, *RP1* 1 year after rewetting and profiling, *R1* 1 year after rewetting, *R10* 10 years after rewetting, *REF* reference site.

**Table 2:** Vegetation cover type and vegetation volume and water table level averages (± SD) at each collar type across the 2016 study period (May–September).

Sector	Type of cover	# Collars	% Cover				Vegetation volume index	Water table level (cm)
			Vascular	Shrubs	Graminoid	Bryophytes		
UNR	Bare (82%)	3	16 (7)	0	7 (7)	0	0	− 18.6 (9.5)
	Herbaceous (18%)	3	40 (10)	0	40 (13)	4 (4)	12.2 (7.6)	− 6.8 (5.3)
RP1	Open water (100%)	6	–	–	–	–	–	43.5 (6.7)
R1	Bare (15%)	3	20 (10)	4 (7)	14 (9)	1 (1)	6.5 (4.9)	5.7 (12.0)
	Herbaceous (66%)	3	47 (20)	5 (6)	43 (19)	2 (3)	19.6 (4.5)	− 0.88 (4.6)
	Shrubs (19%)	3	67 (10)	37 (3)	47 (21)	4 (4)	25.2 (4.5)	− 7.0 (3.8)
R10	Herbaceous (98%)	3	57 (8)	2 (3)	57 (8)	–	14.1 (4.9)	21.0 (7.1)
	Shrubs (2%)	3	77 (8)	23 (6)	75 (5)	–	28.1 (6.6)	20.3 (7.3)
REF	Shrubs (47%)	5	53 (14)	19 (16)	31 (9)	53 (19)	18.1 (6.6)	9.0 (6.8)
	Herbaceous (53%)	4	58 (15)	5 (5)	52 (19)	44 (18)	3.4 (6.6)	9.1 (6.6)

Percentages reported for each cover type is their relative cover across the sector based on vegetation survey plot classifications.

*UNR* unrestored, *RP1* 1 year after rewetting and profiling, *R1* 1 year after rewetting, *R10* 10 years after rewetting, *REF* reference site.

A positive water table level indicates the water is above the peat surface.

**Figure 4:**
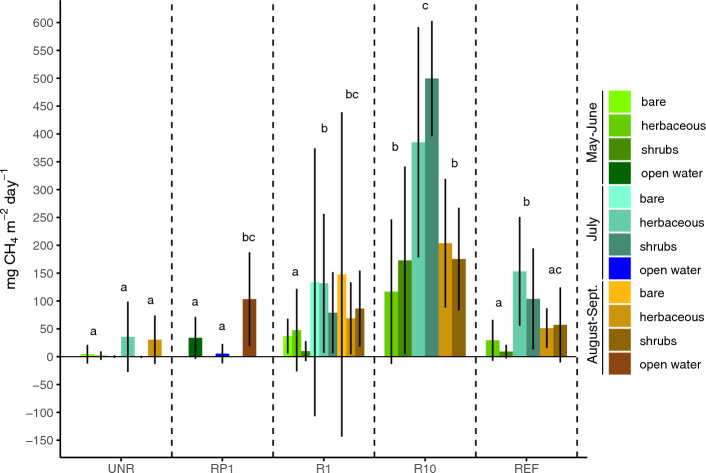
Mean (± SD) CH_4_ fluxes for each for each major vegetation group at an extracted fen in southeastern Manitoba, Canada. Results are sorted according to period of the growing season (May–June, July, and August to September) because of a highly significant interaction between sector and period of the growing season in the CH_4_ linear mixed effects model (see Supplementary Table 4). Within one period, values sharing a letter are not significantly different (Tukey pairwise comparisons, α = 0.05). Error bars represent the standard deviation of the means. Least squares means and 95% confidence intervals for each bar group are found in Supplementary Table 5. *UNR* unrestored, *RP1* 1 year after rewetting and profiling, *R1* 1 year after rewetting, *R10* 10 years after rewetting, *REF* reference site.

Total seasonal CH_4_ fluxes were higher at the restored sectors and REF than at UNR, and seasonal variation in fluxes was influenced by study location, with generally the highest emissions in July (Table 1, Fig. 4, and Supplementary Table 4). Methane fluxes at UNR stayed close to zero during the entire the study period (Table 1 and Fig. 4). Throughout the growing season, R10 was generally characterized by consistently higher CH_4_ fluxes than any other sector, although August and September had less evidence to support differences between R10 and the other sectors (Fig. 4). After summing net study period CO_2_ and CH_4_ exchange values, global warming potential values were positive at UNR and RP1 (Table 1). High CH_4_ emissions considerably reduced the greenhouse gas sequestration potential at R10, with mean decrease of 70% between NEE and GWP.

**Figure 5:**
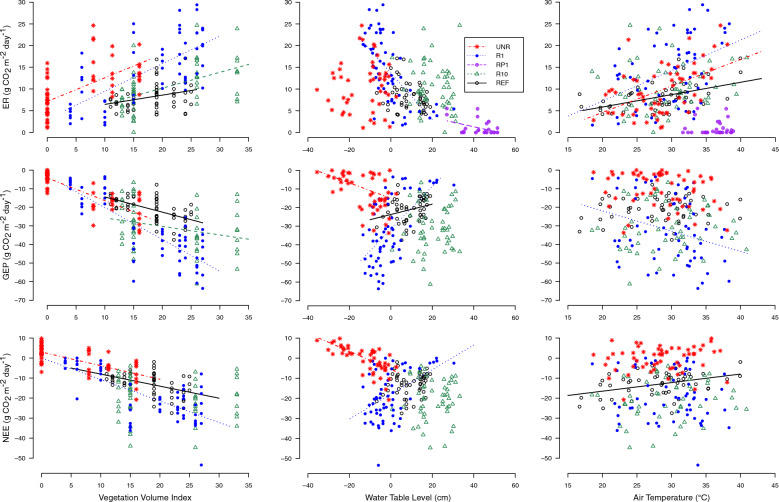
Relationships between ecosystem respiration (ER), gross ecosystem productivity (GEP), and net ecosystem exchange (NEE) with vegetation volume index, water table level, and air temperature under full light conditions (PAR > 1000 μmol m^–2^ s^–1^). Only statistically significant linear regression lines are displayed. Regression equations are given in Supplementary Table 7. Negative fluxes represent carbon uptake into the ecosystem and positive water table level indicates the water is above the peat surface.

Variations in GEP across all sites were related to water table level, vegetation volume, and sector, supported by strong evidence from the linear mixed effect model in this study (Supplementary Table 6; Fig. 5). The interaction between sector and the other factors also had an impact on GEP, including air temperature (Supplementary Table 6). For each sector, a greater volume of vegetation was related to a greater productivity, although the slope of the relation was different between sectors (Fig. 5). The relationship between water table depth and GEP were sector-specific, where higher water table level was related to greater productivity at UNR but was associated with lower productivity rates (R1 and REF) or had no apparent relationship (R10) at the restored sectors and reference site (Fig. 5).

All factors and their interaction with sector were also related to ecosystem respiration (ER) and are supported by strong evidence from the linear mixed effects model (Supplementary Table 6). As with GEP, greater vegetation volume was associated with greater ER, with variation in the slopes between sectors (Fig. 5). Higher water table levels corresponded with lower ER at R1 and RP1 and higher air temperatures were related to higher ER at all sites except R10 (Fig. 5). Steeper slopes for the positive relationship between air temperature and ER occurred at the UNR and R1 sectors compared to the reference site.

**Figure 6:**
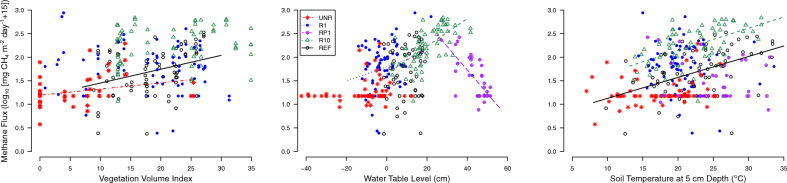
Relationship between methane flux and vegetation volume index, water table level, and soil temperature at 5 cm depth for the experimental sectors and reference site. Only statistically significant linear regression lines are displayed. Regression equations are given in Supplementary Table 7. Positive water table level indicates the water is above the peat surface.

Variations in NEE were also related to all factors and their interaction with sector as supported by strong evidence from the linear mixed effects model (Supplementary Table 6). Higher vegetation volumes led to higher CO_2_ sequestration rates except at R10 where none of the tested variables had apparent relationships with NEE (Fig. 5). There was a significant relationship between NEE and water table level at R1 and UNR, but the relationships had opposite slopes, where higher water table levels were associated with higher CO_2_ sequestration rates at R1 and lower rates at UNR (Fig. 6). A relationship between air temperature and NEE was only supported at REF, with lower temperatures leading to higher CO_2_ sequestration (Fig. 5).

Methane fluxes also had strong evidence for relationships with water table depth, soil temperature, sector, and their interaction, but did not have consistent apparent relationships to vegetation volume (Supplementary Table 6 and Fig. 6). Methane fluxes were related to water table level at R10 and R1 with higher water levels leading to higher CH_4_ fluxes (Fig. 6). RP1 also had a significant relationship, but higher water tables led to lower CH_4_ emissions. Higher soil temperatures resulted in higher CH_4_ emission at R10 and RP1 (Fig. 6).

## Discussion

### Vegetation succession post-rewetting

Peatland vegetation typical of fens was generally able to quickly establish at the study site following rewetting, which is attributed to both the proximity of a natural fen ecosystem and the local hydrological conditions. The site is surrounded by undisturbed open graminoid to forested fen that provided a local source of plant propagules^31,41^. If the site did not have a natural fen adjacent, it is likely that more general wetland species would have established^31^. Hydrological conditions that allow the propagules to move onto the extracted surface are also important for some fen species, and at the study site, the location of the site within a water track likely facilitated seed dispersal^61^ (Fig. 1). Further, like other studies in the region, high water levels likely allowed for wetland and peatland species to establish on fen peat^31,36,62^. Despite the local source of plant propagules and water flow enabling seed dispersal, vegetation did not establish at RP1 due to very high water levels with flattening of the surface microtopography (Table 2). Although we monitored RP1 in only the first season following restoration and in a year that had higher than average precipitation, this suggests that careful management of water levels is needed to ensure that they are appropriate for targeted fen species to establish^53,63,64^ (e.g. *Carex lasiocarpa*).

The vegetation that established in the rewetted sectors also indicates a general trajectory to reference conditions, but vascular vegetation recovered more successfully than bryophyte communities. By 10 years after rewetting, fen species including *C. lasiocarpa* were dominant in the ecosystem (Supplementary Table 3 and Fig. 2). This species frequently forms large colonies and contributes to the early successional stages of regional fen ecosystems^65^. Further, the cover of moss species *C. stellatum* and *S. cossonii* increased to 20% at R10, supporting the idea that rewetting alone can also be used as an effective restoration strategy for bryophytes establishment^29,31,35,36^ (Supplementary Table 3).

Although moss species were able to establish, moss cover 10 years post-rewetting was still three times lower than the reference ecosystem, suggesting that environmental factors may have played a role in limiting bryophyte expansion (Supplementary Table 3). One factor that may have impacted moss growth could be high water tables as the result of episodic flooding that occurs following blockage of drainage ditches^66,67^. The high abundance of *C. lasiocarpa* and the water table depth ranges for each of the sectors supports that the water table is above the ground surface for the majority of the growing season (Fig. 5, Table 2 and Supplementary Table 2), a condition that contributes to stress for many peatland bryophytes^53,63,68^. Many moss species including the dominant moss species at R10, C. *stellatum* and S*. cossonii,* are preferentially found where the water table is below or at the level of the soil surface and therefore would be restricted to the relatively higher points in the microtopography in flooded environments^69^. Differences in nutrient availability between the experimental and reference sites may have also influenced the ability of certain vascular and moss species to establish^11,31,70,71^. However, water chemistry at the experimental sectors and the reference site overlaps significantly, further suggesting that the site was predominantly influenced by groundwater and would therefore support fen species^11^ (Supplementary Table 1). The high abundance of *C. lasiocarpa* may also shade the moss species and produce large amounts of litter, further adding to the limitation of bryophyte expansion^72,73^. Overall, continued monitoring of the site is needed to determine if moss can recover to reference conditions as successional processes continue and a buildup of lower bulk density peat limits episodic flooding^34^.

### Links between environmental factors and carbon cycling post-rewetting

Vegetation establishment was critical for converting a given sector from a net source to sink of C in the 2016 growing season, as supported by the significant relationships between ER, GEP, and NEE within and across all sites with vegetation volume (Figs. 3 and 5, Table 1 and Supplementary Table 6). This is consistent with previous studies showing that restoration and subsequent plant establishment can transform extracted peatlands to growing season CO_2_ sinks only a few years after restoration (e.g. Ref.^24,74,75^). In contrast, when vegetation was not able to establish after rewetting due to very high water levels, such as RP1, the sector was a net growing season source of C (Table 1). RP1 did, however, have lower ER than the unrestored sector with the absence of an aerated layer in the peat profile^75,76^ (Fig. 3). Although ER was decreased with flooding of RP1, some vegetation growth within the unrestored sector meant that they had comparable C release, supporting that flooding alone is unable to counteract C loss, particularly if water level is deep enough to inhibit vegetation establishment^31^ (Tables 1 and 2, Fig. 3). Longer term studies at this site, along with replicate rewetting studies in similar extracted peatlands with exposed minerotrophic peat are needed to assess optimal water table position for carbon sequestration.

Temperature also impacted CO_2_ fluxes across the sectors, where higher temperatures increased ER and subsequently decreased NEE, which was significant at the sector-scale (Fig. 5). A positive relationship of temperature to ER is expected, as higher temperatures generally promote microbial activity up to a certain threshold^77^. High variability in whether individual sites had a significant relationship despite a significant interaction between temperature and sector, however, suggests that sector-scale conditions control the importance of temperature over other factors. These factors include water table and vegetation cover, which can counteract or further increase ER values by controlling aeration and substrate lability in the peat profile^75,78^ (Supplementary Table 6).

Differences in the responses of CO_2_ exchange to water table levels in each sector was likely due to differences in microtopography that impacted local hydrological conditions, and subsequently, vegetation community structure. At the rewetted sectors and the reference site, a higher GEP and NEE was generally related to drier conditions (i.e. a water table below the surface) and higher vegetation volume index (Fig. 5). A higher GEP under drier conditions likely resulted from the shift from sedge-dominated to shrub-dominated cover, which increased vegetation volume index as more plants were able to colonize the surface^79^ (Table 2 and Supplementary Fig. 2). Whether a given sector had a significant relationship with water table depended on their proportion of shrub cover type plots and water levels, where R1 was the only site with a mean water table levels above and below the ground surface and had > 15% shrub-dominated plots and therefore captured the variation between plot types (Table 2). At UNR, the relationship was the opposite, where GEP and NEE increased as conditions were wetter (Fig. 5). Higher GEP as the result of wetter conditions may also be linked to vegetation cover, where wetter portions of the site are within the tolerance limits of the surrounding fen and other vegetation and it is able to colonize those portions of the surface^53^ (Table 2; e.g. *Carex lasiocarpa*).

Differences in plant communities and hydrologic conditions also played an important role in CH_4_ fluxes and subsequently impacted the total C exchanged in the growing season as well as the global warming potential. The sector that was restored 10 years prior to 2016 had the highest CH_4_ emissions of any of the experimental sectors and was also dominated by *Carex lasiocarpa* (R10; Fig. 4, Table 1, and Supplementary Table 3). High percent cover of *C. lasiocarpa* that was inundated for the majority of the 2016 growing season likely contributed to high CH_4_ emissions, as their aerenchyma directly transport CH_4_ to the atmosphere, therefore bypassing the oxidation zone in the water column^80–83^. A lack of evidence for a relationship between CH_4_ flux and vegetation volume at R10 and the other rewetted sites, however, indicates that the presence of vegetation alone cannot predict CH_4_ fluxes (Fig. 6). Instead, species or functional group abundance is likely a more important factor^82,84–86^. Relationships between water table depth and CH_4_ flux were also apparent in all rewetted sectors, with higher CH_4_ emissions associated with higher water table levels until around 30 cm above the surface, where oxidation in the water column and lack of vegetation and high quality substrate likely reduced CH_4_ production and emission^79,81^. Management of the water table position to avoid inundation will thus be important to balance establishing vegetation cover and limiting CH_4_ emissions, especially when graminoid vegetation is dominant and the restoration goal of a project is maximizing the greenhouse gas sequestration function.

Although net C uptake for the growing season was higher at the restored sites with vegetation than at the reference site, interannual variability in environmental variables and the focus on growing season fluxes in this study should be considered when interpreting the C sink strength of peatlands restored using rewetting. High CO_2_ uptake at the rewetted sectors was likely caused by elevated water table during the study period leading to lower heterotrophic respiration rates, and by vegetation establishment following rewetting^24^. Newly restored peatland ecosystems can also initially represent considerable CO_2_ sinks because of an increase in microbial and plant biomass initially that declines through time^87^. Previous studies have also shown that variation in temperature and precipitation each year can cause restored and natural peatland ecosystems to be a source or sink of C^24,33,88^. It is also important to keep in mind that this study only covered four months of the growing season during peak productivity. Losses of CO_2_ through decomposition during the rest of the growing season and cold months are likely to be high given the labile nature of the sedge litter^89^. Therefore, C loss during the rest of the year results in lower net C sequestration in a restored sector on an annual scale with non-growing season NEE comparable to natural sites in previous studies (< 6 g CO_2_ m^–2^ day^–^^24,33^). Therefore, capturing seasonal and interannual variability is needed to fully evaluate long-term restoration success in terms of restoring C dynamics. Nonetheless, establishment of C uptake during the summer months at rates similar to the natural reference fen indicates that rewetting alone on minerotrophic peat shows great promise for restoring C sink function following peat extraction when conditions are favorable for fen vegetation establishment.

## Conclusions

Active rewetting can be an appropriate restoration option for post-extraction fens in North America, inducing a rapid shift in vegetation communities towards natural fen cover. Rewetting and subsequent colonization of former peat extraction sites by vegetation can also lead to the fast return of the growing season C sequestration function. However, before deciding to rely only on rewetting to restore a site with residual fen peat, site managers should ensure that certain conditions are met: (1) a source of propagules should be located near the site and (2) water levels should be monitored and managed with great care (e.g. gradual blocking of the drainage ditches over several years to avoid deep inundation). Further, consideration of non-growing season fluxes is needed to assess the total annual C sink potential of rewetted sites on minerotrophic peat, as they also have high amounts of labile organic C that could be released overwinter via decomposition. To promote bryophyte establishment and to maximize the C sequestration potential by avoiding high CH_4_ emissions, a water level close to the surface should be targeted and deep flooding should be avoided. For the sites where the adjacent pool of species is not composed of fen species, active re-introduction of targeted species should be considered. 

### Supplementary Information

Supplementary Information.

## Data Availability

Data from this study will be made available in Borealis (https://borealisdata.ca) upon publication.
