# Supplementary material for: Rewetting increases vegetation cover and net growing season carbon uptake under fen conditions after peat-extraction in Manitoba, Canada
Source: Sci Rep. 2023 Nov 23;13:20588. doi: 10.1038/s41598-023-47879-y (PMC10667249; doi:10.1038/s41598-023-47879-y)
Supplement: Supplementary file 1 — Supplementary Information. [file 41598_2023_47879_MOESM1_ESM.docx]

# Supplementary Materials for:

**Rewetting increases vegetation cover and net growing season carbon uptake under fen conditions after peat-extraction in Manitoba, Canada**

Laurence Turmel-Courchesne, Marissa A. Davies, Mélina Guêné-Nanchen, Maria Strack, and Line Rochefort

**Corresponding Authors:** Marissa A. Davies (m3davies@uwaterloo.ca); Maria Strack (mstrack@uwaterloo.ca)

This file includes:

Supplementary Figure 1

Supplementary Figure 2

Supplementary Table 1

Supplementary Table 2

Supplementary Table 3

Supplementary Table 4

Supplementary Table 5

Supplementary Table 6

Supplementary Table 7

**Supplementary Figure 1:** Images of experimental sectors and reference sites. **A.** UNR: unrestored; **B.** R1: 1 year after rewetting; **; C.** RP1: 1 year after rewetting and profiling; **D.** R10: 10 years after rewetting; **E.** REF: reference site open graminoid fen area; **F.** REF: reference site forested fen area. Photo credit: L. Turmel-Courchesne.

**Supplementary Figure 2:** Example images of collars categorized as **A.** shrubby and **B.** herbaceous for greenhouse gas exchange measurements from the reference sites (REF). Photo credit: L. Turmel-Courchesne.

**Supplementary Table 1:** Pore water and peat chemistry post-rewetting for the experimental sectors and reference site at the study location. Element units are in mg L^-1^ and mg g^-1^ for pore water and peat respectively. Electrical conductivity (EC) is in μS cm^-1^. N indicates the number of samples. Pore water and soil pH and EC were measured in the field with a handheld device and EC was corrected according to pH and temperature (Hanna Instruments;^1^). The concentration of Ca, Mg, Na, K, and P/PO_4_^3-^ were determined on pore water and peat samples using inductively coupled plasma optical emission spectrometry (ICP-OES, Model 5110, Agilent Technologies) at Université Laval, Québec, Canada. For the pore water samples, Ca, Mg, Na, K, and P/PO_4_^3-^ were extracted for analysis using the method from Amacher, et al. ^2^ and P was mineralized according to Parkinson and Allen ^3^. Peat samples were dried at 40°C for 2 to 3 days prior to analysis and then sieved to < 2 mm. Ca, Mg, Na, and K from peat samples were extracted using a Mehlich 3 extractant and P was extracted with a Bray II extractant. Total N/NH_4_^+^ and N/NO_3_^-^ concentrations in peat and pore water samples were determined by colorimetry via continuous flow injection analysis at the Université Laval, Québec, Canada (QuikChem 8500 Series 2 System, Lachat Instruments). N/NH_4_^+^ and N/NO_3_^-^ were extracted from pore water and peat using the method outlined in Keeney and Nelson ^4^ as well as the normalized QuikChem methods for N/NH_4_^+^ and N/NO_3_^-^ (i.e., 10-107-06-2-B for N/NH_4_+ and 12-107-04-1-F for N/NO_3_^-^; Lachat Instrruments).

| 1. **Water** | | | | | | | | | | | |
| --- | --- | --- | --- | --- | --- | --- | --- | --- | --- | --- | --- |
| **Treatment** | N | pH | EC | Ca | Mg | Na | K | N/NH_4_^+^ | N/NO_3_^-^ | P | PO_4_^3-^ |
| **UNR** | 5 | 6.2  (0.2) | 334  (61) | 35.7  (7.2) | 14.7  (3.6) | 2.1  (3.6) | 4.7  (1.8) | 1.9  (1.5) | 0.20  (0.35) | 0.4  (0.4) | 0.45  (0.29) |
| **RP1** | 8 | 7.9  (0.5) | 231  (44) | 26.5  (4.8) | 11.6  (2.1) | 1.3  (0.2) | 1.2  (0.6) | 0.0  (0.0) | 0.02  (0.01) | 0.1  (0.0) | 0.01  (0.01) |
| **R1** | 5 | 6.5  (0.2) | 424  (143) | 47.5  (15.4) | 19.9  (5.1) | 3.4  (2.2) | 3.5  (0.4) | 0.4  (0.5) | 0.03  (0.01) | 0.2  (0.2) | 0.13  (0.20) |
| **R10** | 5 | 7.5  (0.2) | 259  (35) | 33.9  (6.1) | 12.2  (1.3) | 1.4  (0.3) | 0.8  (0.4) | 0.0  (0.0) | 0.01  (0.00) | 0.1  (0.0) | 0.10  (0.09) |
| **REF - herbaceous** | 4 | 7.1  (0.5) | 270  (42) | 33.1  (7.9) | 12.2  (1.4) | 1.3  (0.2) | 0.5  (0.2) | 0.0  (0.0) | 0.01  (0.01) | 0.2  (0.0) | 0.01  (0.02) |
| **REF - shrubs** | 4 | 7.3  (0.3) | 360  (63) | 42.6  (14.4) | 18.4  (10.6) | 3.1  (1.1) | 1.2  (0.6) | 0.0  (0.0) | 0.01  (0.01) | 0.1  (0.0) | 0.00  (0.01) |
| 1. **Peat** | | | | | | | | | | | |
| **Treatment** | N | pH | EC | Ca | Mg | Na | K | N/NH_4_^+^ | N/NO_3_^-^ | P | PO_4_^3-^ |
| **UNR** | 2 | 6.3  (1.5) | 224  (136) | 18.8  (8.8) | 2.4  (0.5) | 0.0  (0.0) | 0.2  (0.1) | 0.0  (0.0) | 0.12  (0.07) | 0.0  (0.0) | - |
| **RP1** | 0 | - | - | - | - | - | - | - | - | - | - |
| **R1** | 3 | 6.0  (0.4) | 101  (20) | 16.1  (2.8) | 2.6  (0.5) | 0.0  (0.0) | 0.1  0.0 | 0.0  (0.0) | 0.05  (0.03) | 0.0  (0.0) | - |
| **R10** | 3 | 6.7  (0.2) | 123  (57) | 24.8  (0.7) | 2.5  (0.4) | 0.0  (0.0) | 0.4  (0.1) | 0.1  (0.0) | 0.04  (0.01) | 0.1  (0.0) | - |
| **REF - herbaceous** | 2 | 6.7  (0.1) | 137  (25) | 15.8  (0.9) | 1.7  (0.1) | 0.1  (0.0) | 0.6  (0.3) | 0.1  (0.0) | 0.06  (0.01) | 0.0  (0.0) | - |
| **REF - shrubs** | 0 | - | - | - | - | - | - | - | - | - | - |

**Supplementary Table 2:** Mean and standard deviation of daily soil temperature at 5 cm below the ground surface, air temperature, water table position, and photosynthetically active radiation (PAR) during the study period (2016) at the experimental sectors, reference site, and meteorological station. These values were recorded at each site using Onset HOBO Pro v2 and Solinst leveloggers for temperature and water table level respectively. Positive values for water table position indicate that the water table was above the peat surface. UNR: unrestored; RP1: 1 year after rewetting and profiling; R1: 1 year after rewetting; R10: 10 years after rewetting; REF: reference site; STAT: meteorological station.

| **Sector** | **Soil temperature (^o^ C)** | | | |  | **Water table position (cm)** | | | |
| --- | --- | --- | --- | --- | --- | --- | --- | --- | --- |
|  | May-June | July | Aug.-Sept. | Study period average |  | May-June | July | Aug.-Sept. | Study period average |
| UNR | 16.1 (2.4) | 20.0 (1.2) | 18.0 (2.4) | 17.9 (2.6) |  | -14.1 (7.8) | -5.2 (2.5) | -15.0 (12.4) | -11.8 (8.9) |
| RP1 | 21.3 (1.5) | 22.5 (1.1) | 18.8 (2.2) | 20.4 (2.5) |  | 42.5 (5.7) | 49.6 (2.7) | 37.9 (3.4) | 42.6 (6.3) |
| R1 | 18.2 (3.4) | 21.7 (1.0) | 19.4 (2.1) | 19.6 (2.8) |  | - | -5.6 (2.8) | -7.1 (4.0) | -6.5 (3.7) |
| R10 | 16.0 (1.7) | 19.4 (0.9) | 17.7 (1.7) | 17.6 (2.0) |  | 19.9 (6.4) | 29.7 (2.2) | 19.9 (3.6) | 22.5 (6.2) |
| REF | 16.5 (2.0) | 19.4 (1.6) | 21.3 (1.2) | 19.2 (2.6) |  | 9.8 (4.5) | 17.1 (1.6) | 8.8 (2.6) | 11.4 (4.7) |
| STAT | - | - | - | - |  | - | - | - | - |
|  | **Air Temperature (^o^ C)** | | | |  | **PAR (μmol m^-2^ s^-1^)** | | | |
| UNR | - | - | - | - |  | - | - | - | - |
| RP1 | 18.5 (2.9) | 20.5 (2.3) | 18.1 (3.4) | 19.0 (3.2) |  | - | - | - | - |
| R1 | 16.1 (3.7) | 18.9 (3.4) | 19.8 (2.9) | 18.4 (3.7) |  | - | - | - | - |
| R10 | 17.4 (2.9) | 20.6 (2.2) | 18.2 (3.5) | 18.6 (3.2) |  | - | - | - | - |
| REF | 16.1 (2.0) | 18.9 (3.4) | 19.8 (2.9) | 18.4 (3.7) |  | - | - | - | - |
| STAT | 16.6 (3.0) | 19.7 (2.3) | 17.1 (3.3) | 17.7 (3.2) |  | 800 (304) | 838 (270) | 694 (210) | 768 (266) |

**Supplementary Table 3:** Percent cover (mean ± SD) of vegetation species in each experimental sector and the reference site (UNR: unrestored; R1: 1 year after rewetting; R10: 10 years after rewetting; REF: reference site) Species are classified according to their preferential habitat. Plant nomenclature according to Flora of North America Editorial Committee ^5^. Only species with a cover higher than 1% in at least one of the vegetation plots are listed in this table.

| 1. **All Vegetation** | | | | | |
| --- | --- | --- | --- | --- | --- |
|  | | **UNR** | **R1** | **R10** | **REF** |
| Number of vascular plant sampling units (1 m^2^) | | 10 | 35 | 40 | 45 |
| Number of bryophyte sampling units (900 cm^2^) | | 100 | 350 | 400 | 450 |
| Total vegetation cover | | 13 (7) | 26 (11) | 39 (28) | 73 (18) |
| Total vascular plant cover | | 13 (7) | 26 (11) | 28 (8) | 40 (12) |
| Total bryophyte cover | | <1 | 1(1) | 18(18) | 57(19) |
| 1. **Trees and Shrubs** | | | | | |
| **Species** | **Pref. Habitat** | **UNR** | **R1** | **R10** | **REF** |
| *Betula pumila* Linnaeus | Peatland |  | <1 | 1 (3) | 7 (8) |
| *Dasiphora fruticosa* (Linnaeus) Rydberg | Facultative |  | <1 | <1 | 5 (8) |
| *Larix laricina* (Du Roi) K. Koch | Facultative |  | <1 |  | 6 (11) |
| *Populus balsamifera* Linnaeus | Facultative |  | 1 (1) |  |  |
| *Salix bebbiana* Sargent | Facultative |  | 1 (1) | <1 |  |
| *Salix discolor* Muhlenberg | Facultative |  | 1 (2) | 1 (1) |  |
| *Salix pedicellaris* Pursh | Peatland |  |  | <1 | 1 (2) |
| *Salix serissima* (L.H. Bailey) Fernald | Wetland |  | 2 (3) | <1 |  |
| *Thuja occidentalis*Linnaeus | Facultative |  |  |  | 2 (6) |
| **TOTAL** | - | <1 | 8(7) | 3(4) | 18(13) |
| 1. **Ericaceae** | | | | | |
| **Species** | **Pref. Habitat** | **UNR** | **R1** | **R10** | **REF** |
| *Andromeda polifolia* Linnaeus | Peatland |  | <1 | 1 (1) | 1 (2) |
| *Rhododendron groenlandicum*(Oeder) Kron & Judd | Wetland |  |  | <1 | 2 (6) |
| *Vaccinium oxycoccos*Linnaeus | Peatland |  | <1 | <1 | 2 (4) |
| **TOTAL** | - |  |  | 1 (1) | 5 (7) |
| 1. ***Carex*** | | | | | |
| **Species** | **Pref. Habitat** | **UNR** | **R1** | **R10** | **REF** |
| *Carex aquatilis* Wahlenberg | Peatland | <1 | <1 | 2 (3) | 3 (5) |
| *Carex chordorrhiza*Linnaeus | Peatland |  |  | 1 (2) | <1 |
| *Carex interior*L.H. Bailey | Peatland | <1 | 3 (4) | <1 |  |
| *Carex lasiocarpa*Ehrhart | Peatland | 1 (2) | 1 (1) | 17 (10) | 9 (7) |
| *Carex tenuiflora*Wahlenberg | Peatland |  | 1 (2) | <1 | <1 |
| *Carex trisperma*Dewey | Peatland | <1 | 1 (4) |  |  |
| **TOTAL** | - | 2 (2) | 8 (6) | 22 (11) | 12 (6) |

**Supplementary Table 3** (continued).

| 1. **Herbaceous** | | | | | |
| --- | --- | --- | --- | --- | --- |
| **Species** | **Pref. Habitat** | **UNR** | **R1** | **R10** | **REF** |
| *Agrostis scabra* Willd. | Facultative | 4 (5) | 2 (2) |  |  |
| *Bidens cernua*Linnaeus | Wetland | 1 (2) | <1 |  |  |
| *Calamagrostis stricta*(Timm) Koeler | Facultative |  | <1 | 1 (1) | <1 |
| *Cirsium arvense*(Linnaeus) Scopoli | Other | 1 (1) | <1 |  |  |
| *Comarum palustre*Linnaeus | Peatland |  |  | <1 | 1 (2) |
| *Eleocharis acicularis*(Linnaeus) Roemer & Schultes | Wetland |  | 1 (4) |  |  |
| *Epilobium ciliatum*Rafinesque | Facultative | 1 (1) | <1 | <1 |  |
| *Equisetum fluviatile*Linnaeus | Wetland | <1 | <1 | 1 (1) | <1 |
| *Hordeum jubatum*Linnaeus | Other | 4 (4) | <1 |  |  |
| *Juncus tweedyi*Rydberg | Wetland |  | 1 (3) |  |  |
| *Maianthemum trifolium*(Linnaeus) Sloboda | Wetland |  |  | <1 | 1(3) |
| *Menyanthes trifoliata* Linnaeus | Peatland |  |  | 2 (3) | 5 (8) |
| *Persicaria pensylvanica*(Linnaeus) M. Gómez | Facultative |  |  |  | 1 (3) |
| *Rubus arcticus*Linnaeus | Peatland |  |  | <1 | 1 (2) |
| *Sarracenia purpurea*Linnaeus | Peatland |  |  |  | 1 (2) |
| *Scirpus cyperinus*(Linnaeus) Kunth | Wetland |  | 1 (3) |  |  |
| **TOTAL** | - | 14 (5) | 18 (12) | 7 (6) | 13 (10) |
| 1. **Bryophytes** | | | | | |
| **Species** | **Pref. Habitat** | **UNR** | **R1** | **R10** | **REF** |
| *Aulacomnium palustre* (Hedw.) Schwägr. | Peatland |  | <1 | <1 | 2 (4) |
| *Brachythecium acutum*(Mitt.) Sull. | Peatland |  |  |  | 4 (12) |
| *Ptychostomum pseudotriquetrum*(Hedw.) J.R. Spence & H.P. Ramsay ex Hol. & N. Pedersen | Peatland | <1 | <1 | 1 (2) |  |
| *Calliergon giganteum*(Schimp.) Kindb. | Wetland |  |  |  | 1 (4) |
| *Straminergon stramineum* (Dicks. ex Brid.) Hedenäs | Peatland |  |  |  | <1 |
| *Campylium stellatum*(Hedw.) C.E.O. Jensen | Peatland |  | <1 | 12 (12) | 18 (13) |
| *Climacium dendroides*(Hedw.) F. Weber & D. Mohr | Other |  |  |  | <1 |
| *Dicranum undulatum*Bridel | Peatland |  |  |  | 1 (3) |
| *Pleurozium schreberi*(Willd. ex Brid.) Mitten | Other |  |  |  | 1 (5) |
| *Scorpidium cossonii* (Schimp.) Hedenäs | Wetland |  | <1 | 4 (6) | 22 (15) |
| *Scorpidium scorpioides*(Hedw.) Limpr. | Peatland |  |  | <1 | 1 (4) |
| *Sphagnum angustifolium* (Warnst.) C.E.O. Jensen | Peatland |  |  |  | 1 (5) |
| *Sphagnum fuscum*(Schimp.) H. Klinggr. | Peatland |  |  |  | 3 (13) |
| *Sphagnum russowii*Warnst | Peatland |  |  |  | 1 (6) |
| *Sphagnum warnstorfii* Russow | Peatland |  |  |  | 1 (4) |
| **TOTAL** | - | <1 | <1 | 18 (18) | 57 (19) |

**Supplementary Table 4:** Impacts of rewetting strategy and portion of the study period on CO_2_ and CH_4_ fluxes from linear mixed effect models. Rewetting strategy refers to the experimental sectors and reference site and the period refers to different portions of the growing season, i.e., May-June, July, and August-September.

| **Flux component** | **Fixed effects** | **F _num, den_** | **p** |
| --- | --- | --- | --- |
| Ecosystem respiration (ER) |  |  |  |
|  | Period | F _2, 226_ = 8.9 | 0.0002 |
|  | Sector | F _4, 34_ = 13.9 | <0.0001 |
|  | Period*Sector | F _8, 226_ = 13.9 | 0.0027 |
|  | Intercept | F_1, 226_ = 224.3 | <0.0001 |
| Gross ecosystem productivity (GEP) | |  |  |
|  | Period | F _2, 198_ = 13.5 | <0.0001 |
|  | Sector | F _3, 29_ = 6.4 | 0.0019 |
|  | Period*Sector | F _6, 198_ = 13.5 | 0.0543 |
|  | Intercept | F_1, 198_ = 187.4 | <0.0001 |
| Net ecosystem exchange (NEE) | |  |  |
|  | Period | F _2, 198_ = 3.7 | 0.026 |
|  | Sector | F _3, 29_ = 10.1 | 0.0001 |
|  | Period*Sector | F _6, 198_ = 1.3 | 0.27 |
|  | Intercept | F_1, 198_ = 129.8 | <0.0001 |
| CH_4_ Fluxes |  |  |  |
|  | Period | F _2, 267_ = 37.8 | <0.0001 |
|  | Sector | F _4, 31_ = 22.8 | <0.0001 |
|  | Period*Sector | F _8, 267_ = 18.8 | <0.0001 |
|  | Intercept | F_1, 267_ = 2580.6 | <0.0001 |

**Supplementary Table 5:** Least squares mean CO_2_ fluxes and CH_4_ emissions for the study period of all collars (95% confidence interval in brackets). GEP: gross ecosystem productivity, ER: ecosystem respiration, NEE: net ecosystem exchange. GEP and NEE are measured at photon flux density of photosynthetically active radiation greater than 1000 μmol m^-2^ s^-1^. Negative values represent uptake by the ecosystem. UNR: unrestored sector; R1: rewetted sector with one growing season since restoration; R10: rewetted sector with 10 growing seasons since restoration; REF: reference site; RP1: rewetted and profiled sector with one growing season since restoration. Statistical significance among groups are indicated in Figures 3 and 4 of the main text.

| **Sector** | **CO_2_ (g CO_2_ m^-2^d^-1^)** | | |  | **CH_4_ (mg CH_4_ m^-2^d^-1^)** | | |
| --- | --- | --- | --- | --- | --- | --- | --- |
|  | **ER** | **GEP** | **NEE** |  | **May-June** | **July** | **Aug.-Sept.** |
| UNR | 10.5  [7.4, 13.6] | -12.7  [-20.9, -4.5] | -2.1  [-7.5, 3.3] |  | 2.2  [-3.2, 10.1] | 3.5  [-2.8, 12.9] | 3.0  [-3.1, 12.1] |
| RP1 | 1.16  [0, 4.0] | - | - |  | 21.4  [5.7, 49.0] | 1.8  [-4.4, 11.5] | 82.2  [43.6, 146.0] |
| R1 | 14.2  [11.5, 16.9] | -32  [-38.9, -25.1] | -17.7  [-22.2, -13.1] |  | 15.4  [4.7, 32.0] | 69.3  [43.0, 107.5] | 64.9  [39.6, 101.8] |
| R10 | 10.8  [7.6, 13.9] | -31.3  [-62.6, -22.4] | -20.5  [-26.5, -14.6] |  | 93.5  [57.0, 148.6] | 416.5  [257.4, 668.8] | 164.0  [98.4, 267.4] |
| REF | 7.9  [5.7, 10.0] | -20.9  [-27.0, -14.7] | -13.0  [-17.3, -8.8] |  | 8.7  [1.0, 20.2] | 107.9  [69.4, 163.8] | 31.1  [16.2, 53.2] |

**Supplementary Table 6:** Controls over CO_2_ and CH_4_ fluxes from linear mixed effect models. RP1: 1 year after rewetting and profiling; Others: all other locations (i.e., UNR: unrestored; R1: 1 year after rewetting; R10: 10 years after rewetting; REF: reference site), WTL: water table level (cm), Veg_Vol: vegetation volume index; Tair: air temperature (°C); T5: soil temperature at 5 cm (°C).

| **Flux component** | **Fixed effects** | **F _num, den_** | **p** | **R^2^GLMM** |
| --- | --- | --- | --- | --- |
| Ecosystem respiration (ER) |  |  |  |  |
| RP1 | WTL | F_1, 25_ = 7.16 | 0.0129 | 0.19 |
|  | Intercept | F_1, 25_ = 12.71 | 0.0015 |  |
| Others | WTL | F _1, 181_ = 51.2 | <0.0001 | 0.42 |
|  | Veg_Vol | F _1, 22_ = 72.65 | <0.0001 |  |
|  | Tair | F _1, 181_ = 68.75 | <0.0001 |  |
|  | Sector | F _3, 22_ = 8.62 | 0.0006 |  |
|  | Sector *WTL | F _3, 181_ = 2.1 | 0.102 |  |
|  | Sector * Veg_Vol | F _3, 22_ = 2.93 | 0.0056 |  |
|  | Sector *Tair | F _3,181_ = 6.93 | 0.0002 |  |
|  | Intercept | F_1, 81_ = 778.24 | <0.0001 |  |
| Gross ecosystem productivity (GEP) | |  |  |  |
|  | WTL | F _1, 181_ = 33.36 | <0.0001 | 0.51 |
|  | Veg_Vol | F _1, 22_ = 155.78 | <0.0001 |  |
|  | Tair | F _1, 181_ = 2.32 | 0.13 |  |
|  | Sector | F _1, 22_ = 39.85 | <0.0001 |  |
|  | Sector *WTL | F _3, 181_ = 7.42 | 0.0001 |  |
|  | Sector * Veg_Vol | F _3, 22_ = 7.85 | 0.001 |  |
|  | Sector *Tair | F _3, 181_ = 4.99 | 0.0024 |  |
|  | Intercept | F_1, 181_ = 965.3 | <0.0001 |  |
| Net ecosystem exchange (NEE) |  |  |  |  |
|  | WTL | F_1, 177_ = 32.5 | <0.0001 | 0.27 |
|  | Veg_Vol | F_1, 22_ = 407.6 | <0.0001 |  |
|  | Tair | F _1, 177_ = 15.3 | 0.0001 |  |
|  | Sector | F _1, 22_ = 12.5 | 0.0001 |  |
|  | Sector *WTL | F _3,177_ = 15.0 | <0.0001 |  |
|  | Sector * Veg_Vol | F _3, 22_ = 11.3 | 0.0001 |  |
|  | Sector *Tair | F _3,177_ = 4.2 | 0.007 |  |
|  | Intercept | F_1, 177_ =651.4 | <0.0001 |  |
| CH_4_ Fluxes |  |  |  |  |
| RP1 | WTL | F_1, 31_ = 45.5 | <0.0001 | 0.54 |
|  | Intercept | F_1, 31_ = 301.7 | <0.0001 |  |
| Others | WTL | F_1, 211_ = 9.9 | <.0001 | 0.49 |
|  | Veg_Vol | F_1, 211_ = 1.3 | 0.25 |  |
|  | T5 | F _1, 211_ = 34.9 | <0.0001 |  |
|  | Sector | F _3, 26_ = 28.4 | <0.0001 |  |
|  | Sector *WTL | F _3, 211_ = 23.2 | <0.0001 |  |
|  | Sector * Veg_Vol | F _3, 211_ = 2.03 | 0.11 |  |
|  | Sector *T5 | F _3, 211_ = 18.1 | <0.0001 |  |
|  | Intercept | F_1, 211_ =2109.5 | <0.0001 |  |

**Supplementary Table 7:** Linear regression equations for each sector and carbon component within the linear mixed effects model. Units for ecosystem respiration (ER), gross ecosystem productivity (GEP), and net ecosystem productivity (NEE) are g CO_2_ m^-2^d^-1^and methane (CH_4_) is mg CH_4_ m^-2^d^-1^. UNR: unrestored sector; R1: rewetted sector with one growing season since restoration; R10: rewetted sector with 10 growing seasons since restoration; REF: reference site; RP1: rewetted and profiled sector with one growing season since restoration.

| **Sector** | **Y** | **X** | **Equation** | **F** | **p** |
| --- | --- | --- | --- | --- | --- |
| UNR | ER | Vegetation volume index | y = 0.54x + 7.1 | 33.7 | <0.0001 |
|  |  | Water table level (cm) | y = 0.04x + 11.3 | 0.2 | 0.6941 |
|  |  | Air temperature (°C) | y = 0.61x - 7.6 | 19.1 | <0.0001 |
|  | GEP | Vegetation volume index | y = -1.22x - 4.2 | 130.7 | <0.0001 |
|  |  | Water table level (cm) | y = -0.51x -18.0 | 16.0 | 0.0002 |
|  |  | Air temperature (°C) | y = -0.43x + 2.1 | 2.8 | 0.1008 |
|  | NEE | Vegetation volume index | y = -0.68x + 3.0 | 51.0 | <0.0001 |
|  |  | Water table level (cm) | y = -0.48x - 6.7 | 44.0 | <0.0001 |
|  |  | Air temperature (°C) | y = 0.17x - 5.2 | 0.9 | 0.3394 |
|  | CH_4_ | Vegetation volume index | y = 0.01x + 1.2 | 7.1 | 0.0093 |
|  |  | Water table level (cm) | y = 0.00x + 1.3 | 1.5 | 0.2220 |
|  |  | Soil temperature at 5 cm (°C) | y = 0.01x +1.1 | 2.6 | 0.1089 |
| R1 | ER | Vegetation volume index | y = 0.64x + 3.1 | 53.7 | <0.0001 |
|  |  | Water table level (cm) | y = -0.54x + 13.7 | 41.4 | <0.0001 |
|  |  | Air temperature (°C) | y = 0.70x - 6.8 | 13.08 | 0.0006 |
|  | GEP | Vegetation volume index | y = -1.71x - 3.1 | 116.0 | <0.0001 |
|  |  | Water table level (cm) | y = 1.15x - 31.7 | 34.0 | <0.0001 |
|  |  | Air temperature (°C) | y = -1.09x | 5.4 | 0.0233 |
|  | NEE | Vegetation volume index | y = -1.07x | 65.9 | <0.0001 |
|  |  | Water table level (cm) | y = 0.62x - 18.0 | 15.8 | 0.0002 |
|  |  | Air temperature (°C) | y = -0.39x - 6.9 | 1.3 | 0.2564 |
|  | CH_4_ | Vegetation volume index | y = 0.00x + 1.8 | 0.4 | 0.5115 |
|  |  | Water table level (cm) | y = 0.01x + 1.8 | 5.2 | 0.0257 |
|  |  | Soil temperature at 5 cm (°C) | y = -0.02x + 2.2 | 1.5 | 0.2250 |

**Supplementary Table 7** (continued).

| **Sector** | **Y** | **X** | **Equation** | **F** | **p** |
| --- | --- | --- | --- | --- | --- |
| R10 | ER | Vegetation volume index | y = 0.37x + 2.9 | 19.1 | <0.0001 |
|  |  | Water table level (cm) | y = -0.10x + 12.6 | 1.1 | 0.3024 |
|  |  | Air temperature (°C) | y = 0.10x + 7.4 | 0.5 | 0.4830 |
|  | GEP | Vegetation volume index | y = -0.46x - 21.1 | 4.1 | 0.0466 |
|  |  | Water table level (cm) | y = 0.09x - 32.5 | 0.1 | 0.7041 |
|  |  | Air temperature (°C) | y = 0.09x - 33.2 | 0.1 | 0.7976 |
|  | NEE | Vegetation volume index | y = -0.09x - 18.2 | 0.2 | 0.6307 |
|  |  | Water table level (cm) | y = -0.01x - 19.9 | 0.0 | 0.9466 |
|  |  | Air temperature (°C) | y = 0.19x - 25.8 | 0.5 | 0.4990 |
|  | CH_4_ | Vegetation volume index | y = 0.01x + 2.0 | 2.2 | 0.1412 |
|  |  | Water table level (cm) | y = 0.03x + 1.6 | 43.8 | <0.0001 |
|  |  | Soil temperature at 5 cm (°C) | y = 0.05x + 1.2 | 28.9 | <0.0001 |
| REF | ER | Vegetation volume index | y = 0.21x + 4.3 | 4.8 | 0.0330 |
|  |  | Water table level (cm) | y = -0.13x + 9.4 | 3.9 | 0.0534 |
|  |  | Air temperature (°C) | y = 0.29x + 0.2 | 16.5 | 0.0002 |
|  | GEP | Vegetation volume index | y = -0.82x - 6.3 | 21.2 | <0.0001 |
|  |  | Water table level (cm) | y = 0.28x - 23.7 | 4.3 | 0.0430 |
|  |  | Air temperature (°C) | y = 0.14x - 25.3 | 0.7 | 0.3934 |
|  | NEE | Vegetation volume index | y = -0.60x - 2.0 | 12.8 | 0.0008 |
|  |  | Water table level (cm) | y = 0.15x - 14.4 | 1.5 | 0.2251 |
|  |  | Air temperature (°C) | y = 0.43x - 25.1 | 9.8 | 0.0030 |
|  | CH_4_ | Vegetation volume index | y = 0.04x + 1.0 | 13.7 | 0.0004 |
|  |  | Water table level (cm) | y = 0.01x + 1.6 | 1.7 | 0.1990 |
|  |  | Air temperature (°C) | y = 0.04x + 0.7 | 20.0 | <0.0001 |
| RP1 | ER | Water table level (cm) | y = -0.11x + 5.6 | 7.2 | 0.0120 |
|  |  | Air temperature (°C) | y = 0.00x + 1.1 | 0.0 | 0.9266 |
|  | CH_4_ | Water table level (cm) | y = -0.05x + 3.7 | 35.0 | <0.0001 |
|  |  | Soil temperature at 5 cm (°C) | y = -0.02x + 2.1 | 2.6 | 0.1182 |

**References:**

1 Sjörs, H. On the Relation between Vegetation and Electrolytes in North Swedish Mire Waters. *Oikos* **2**, 241-258, doi:10.2307/3564795 (1950).

2 Amacher, M. C., Henderson, R. E., Breithaupt, M. D., Seale, C. L. & LaBauve, J. M. Unbuffered and Buffered Salt Methods for Exchangeable Cations and Effective Cation-Exchange Capacity. *Soil Science Society of America Journal* **54**, 1036-1042, doi:10.2136/sssaj1990.03615995005400040018x (1990).

3 Parkinson, J. A. & Allen, S. E. A wet oxidation procedure suitable for the determination of nitrogen and mineral nutrients in biological material. *Communications in Soil Science and Plant Analysis* **6**, 1-11, doi:10.1080/00103627509366539 (1975).

4 Keeney, D. R. & Nelson, D. W. Nitrogen-inorganic forms in *Methods of Soil Analysis Part 2 (2nd Ed.)* (eds A. L. Page, R. H. Miller, & D. R. Keeney) 643-698 (American Society of Agronomy and Soil Sceince Society of America, 1982).

5 Flora of North America Editorial Committee. *Flora of North America North of Mexico 22+ vols.*, <<http://floranorthamerica.org/Main_Page>> (1993+).
